# Identification of *Puccinia striiformis* races from the spring wheat crop in Xinjiang, China

**DOI:** 10.3389/fpls.2023.1273306

**Published:** 2023-10-03

**Authors:** Jinbiao Ma, Muhammad Awais, Li Chen, Hong Yang, Hanlin Lai, Yuyang Shen, Huiqing Wang, Guangkuo Li, Haifeng Gao

**Affiliations:** ^1^ State Key Laboratory of Desert and Oasis Ecology, Key Laboratory of Ecological Safety and Sustainable Development in Arid Lands, Xinjiang Institute of Ecology and Geography, Chinese Academy of Sciences, Urumqi, China; ^2^ State Key Laboratory of Crop Stress Biology for Arid Areas, College of Plant Protection, Northwest A&F University, Shaanxi, Xianyang, China; ^3^ Institute of Plant Protection, Xinjiang Academy of Agricultural Sciences/Key Laboratory of Integrated Pest Management on Crop in Northwestern Oasis, Ministry of Agriculture and Rural Affairs, Urumqi, China; ^4^ College of Agriculture, Xinjiang Agricultural University/Key Laboratory of the Pest Monitoring and Safety Control of Crops and Forests, Urumqi, China; ^5^ College of Life Science, Xinjiang Agricultural University, Urumqi, China; ^6^ Xinjiang Plant Protection Station, Department of Xinjiang Agriculture, Urumqi, China

**Keywords:** wheat stripe rust, race identification, virulence factor, spring wheat, China epidemic regions

## Abstract

Stripe rust, caused by *Puccinia striiformis* f. sp. *tritici* (*Pst*), is a foliar disease that affects both winter and spring wheat crops in Xinjiang, China, which is linked to Central Asia. Race identification of *Pst* from spring wheat in Xinjiang was not done before. In this study, a total of 216 isolates were recovered from stripe rust samples of spring wheat in the region in 2021 and multiplied using the susceptible cultivar Mingxian 169. These isolates were tested on the Chinese set of 19 wheat differential lines for identifying *Pst* races. A total of 46 races were identified. Races *Suwon-11-1*, *Suwon11-12*, and *CYR32* had high frequencies in the spring wheat region. The frequencies of virulence factors on differentials “Fulhard” and “Early Premium” were high (**>**95%), whereas the virulence factor to differential “*Triticum spelta* var. Album” (*Yr5*) was not detected, while virulence to other differentials showed variable frequency within different counties. The predominant races in winter wheat in the same season were also detected from spring wheat cultivars, indicating Pst spreading from winter wheat to spring wheat crops. Deploying resistance genes in spring and winter wheat cultivars is critical for control stripe rust.

## 1 Introduction

Wheat (*Triticum* spp.) is an important cereal crop worldwide. Wheat production must increase to keep pace with continued population growth. Various factors threaten wheat production, including biotic stresses (Singh et al., 2022). Stripe rust, also known as yellow rust (*Yr*), is one of the most destructive diseases of wheat and can cause 100% yield loss with susceptible cultivars and favorable environmental conditions (Begum et al., 2014).

The pathogen of wheat stripe rust, *Puccinia striiformis* f. sp. *tritici* (*Pst*), is highly aggressive and evolves quickly, and new races can overcome race-specific resistance in wheat cultivars and cause severe epidemics (Chen et al., 2010). Thus, it is crucial to identify new races and to develop wheat cultivars with effective and durable resistance to prevent potential disease epidemics (Elbasyoni et al., 2019). To date, 85 Yr genes for stripe rust resistance have been formally designated (*Yr1* to *Yr85*), and more than 100 temporarily named Yr genes or quantitative trait loci (QTL) have been reported (McIntosh et al., 2020; Feng et al., 2023).

The stripe rust fungus is notorious for its capability of long-distance dispersal in the world. Recently, high genetic diversity and recombination populations were reported in China’s north and south epidemic regions. Moreover, these epidemic regions provide inoculum to cause epidemics in other regions of the country (Awais et al., 2022). China has been a primary focus of stripe rust for researchers after the Himalayan region was found as a center of diversity for the worldwide *Pst* populations (Ali et al., 2014).

Disease dynamics involve complex interactions among the host, pathogen, and environment (Newlands, 2018). The vast region of Xinjiang in northwestern China is important for stripe rust epidemics. As it is located in the potential center of diversity (Ali et al., 2014; Awais et al., 2022), the region links other Chinese epidemic regions to Central Asia (Chen et al., 2023). Also, both winter and spring wheat crops grown in this region and mild summer temperatures provide a long season of host plants and favorable environmental conditions for *Pst* to infect, develop, and survive. A recent study (Chen et al., 2023) was conducted to identify *Pst* races identification from winter wheat crops, but the races in spring wheat crops were not studied. The previous history of races on winter and spring wheat hosts is crucial for breaking the life cycle of *Pst*. Furthermore, identifying new stripe rust pathotypes creates an urgent need to develop new stripe rust-resistant lines.

Among different races identified in China using Chinese differential lines, the CYR32 is one of the most predominant races. It was first identified in red Abbondanza in Huangzhong, Qinghai Province, in 1991 (Wan et al., 2003). In 2009, a new Pst race G22 was virulent to cultivars Guinong and 92R lines, Moro, and Chuanmai 42 having resistance genes Yr10 and Yr26 (Liu et al., 2010), which were also virulent to cultivars rapidly spread to other regions (Liu et al., 2015). Races G22-9 (CYR34), CYR32, and CYR33 were China’s most aggressive and predominant races (Liu et al., 2017; Bai et al., 2018; Huang et al., 2020).

Spring wheat in different parts of China is mainly grown in higher latitude or in the higher-elevation areas of Inner Mongolia, Heilongjiang, Qinghai, Ningxia, Xinjiang, Hebei, Tianjin, Shanxi, Gansu, and Tibet, and some small areas in Liaoning and Jilin province (Li et al., 2019). It has some serious drawbacks, especially its susceptibility to most diseases (Sears and Miller, 1985). One of the major reasons is the small breeding and research effort as spring wheat has a relatively small planting area compared to winter wheat. Another reason is that the environmental conditions during the spring wheat growing season is more suitable for disease infection.

This study aimed to identify races of *Pst* from the spring wheat crop of Xinjiang and determine the role of spring and winter wheat crops in the region for the survival of the pathogen, and to determine virulence frequencies of *Pst* in the spring wheat region to help deploy specific resistant cultivars to minimize future epidemics.

## 2 Materials and methods

### 2.1 Stripe rust collection

A total of 216 samples of wheat stripe rust were collected from spring wheat in Gongliu (GL), Nileke (NLK), Tekesi (TKS), Xinyuan (XY), and Zhaosu (ZS) in Yili, Xinjiang, China in the stripe rust surveys at the end of July, during cropping season of 2021 (**Supplementary Table 1**). Samples were collected from different fields at a minimum distance of 15–20 km following the protocol developed by Ali and Hodson (2017). The collected samples were put inside moisture-absorbent bags and labeled with specific sample codes, disease severity, GPS coordinates, crop growth stage, and cultivar information. The sample bags were placed on a table at room temperature overnight for drying and then put in a desiccator with silica at 4°C for later use.

### 2.2 Urediniospore multiplication

The leaf samples were moisturized and put on wet filter papers in petri dishes at 10°C for 10 h for urediniospore production. Urediniospores from individual uredinia were transferred onto a leaf of a susceptible wheat cultivar (Mingxian 169) 10–15 days after planting using a sterilized needle. The inoculated seedlings were sprayed with water, covered with moisturized plastic sheets to avoid contamination, kept in a dew chamber at 10 to 13°C for 24 h in darkness, and then placed in a growth chamber under a day/night thermoperiod of 17/13°C with a photoperiod of 16 h. Fifteen days after inoculation, urediniospores were collected into test tubes. The fresh urediniospores were used to inoculate Mingxian 169 seedlings to produce enough urediniospores. Urediniospores were kept in a desiccator in a refrigerator during the multiplication and stored in a −20°C freezer until further tests. If urediniospores from the freezer were needed for multiplication, the urediniospores were heat-shocked for 2 min by submerging the vials containing urediniospores in warm water (approximately 50°C). For each sample, spores from one uredinium were used to establish an isolate to represent the sample, and therefore, a total of 216 isolates were obtained for race identification.

### 2.3 Race identification

The set of 19 Chinese differential lines were used for identifying races of *Pst*, as described by Zhan et al. (2016). These differential lines carry single and multiple known and unknown *Yr* genes (**Supplementary Table 2**). Seeds of these differential lines, together with Mingxian-169 as a susceptible control, were planted in plastic pots with five lines in each pot. Seedlings (10 to 15 days old) were used for inoculation. Fresh uredospore were mixed with talcum powder at a ratio of 1:30 in a 15-mL centrifuge tube, and the mixture was shaken gently onto seedlings of the differential lines. The inoculated seedlings were kept in the dew chamber for 24 h and then grown in the growth chambers under the conditions as described above. Fifteen days after inoculation, infection type was recorded for each differential using a 0–9 scale (Line and Qayoum, 1992). Infection types 0–6 were considered avirulent and infection types 7–9 were considered virulent (Wan and Chen, 2014). The differential test was repeated to validate the infection type data.

### 2.4 Data analysis

The virulence pattern of each isolate was used to identify the race. The virulence profile of isolates was compiled into an Excel sheet along with previously identified races (Zhan et al., 2016; Chen et al., 2023). Diversity for race and virulence in *P. striiformis* populations sampled from the Xinjiang region was calculated based on the Simpson diversity index “1-D” (Simpson, 1949). The frequencies of races and virulence factors were calculated for each location and the surveyed region. Cluster-based analysis was conducted based on the virulence profiles to assess the relationships of the isolates from different locations using the Ward method (1963) (Ward, 1963).

## 3 Results

### 3.1 Races and diversity

**Figure 1:**
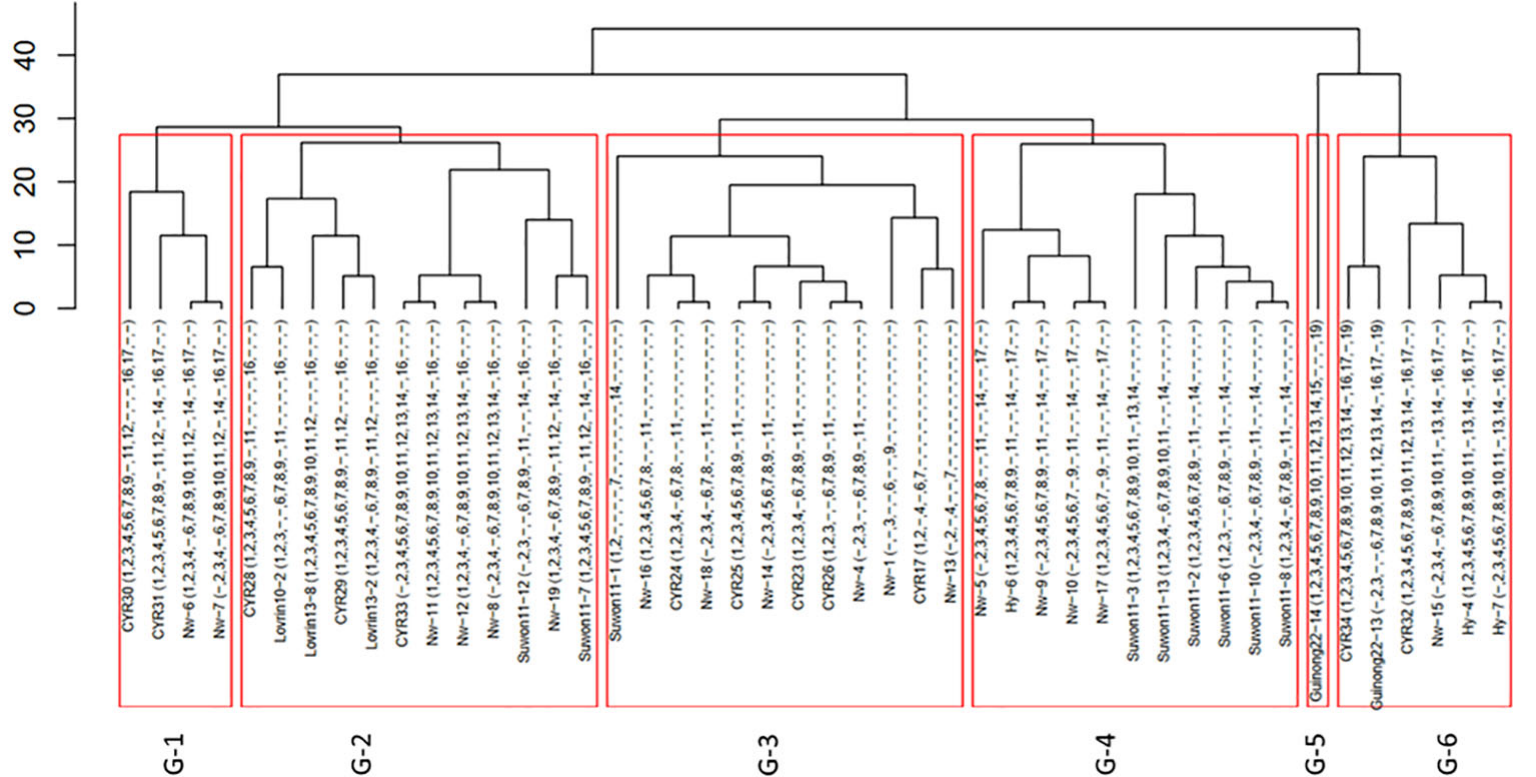
Clustering of 46 races of *Puccinia striiformis* f. sp. *tritici* collected from the spring wheat epidemic region in Xinjiang, China.

From the 216 isolates, 46 *Pst* races were identified, including 29 previously identified races and 17 new races (**Table 1**). As the new races had low frequencies, they were named with temporary numbers with the prefix of Nw standing for northwest. These races were clustered into six groups based on their virulence profiles (**Figure 1**).

**Table 1:** Races of *Puccinia striiformis* f. sp. *tritici* from spring wheat in Xinjiang, China in 2021.

Race	Virulence profile	GL	NLK	TKS	XY	ZS	Overall population
***CYR17***	1,2,-,4,-,6,7,-,-,-,-,-,-,-,-,-,-,-,-	–	3.03	–	–	2.63	0.93
***CYR23***	1,2,3,4,-,6,7,8,9,-,11,-,-,-,-,-,-,-,-	2.08	3.03	–	–	–	0.93
***CYR24***	1,2,3,4,-,6,7,8,-,-,11,-,-,-,-,-,-,-,-	2.08	3.03	–	–	5.26	1.85
***CYR25***	1,2,3,4,5,6,7,8,9,-,11,-,-,-,-,-,-,-,-	2.08	–	–	–	2.63	0.93
***CYR26***	1,2,3,-,-,6,7,8,9,-,11,-,-,-,-,-,-,-,-	–	3.03	1.19	–	–	0.93
***CYR28***	1,2,3,4,5,6,7,8,9,-,11,-,-,-,-,16,-,-,-	2.08	3.03	3.57	–	5.26	3.24
***CYR29***	1,2,3,4,5,6,7,8,9,-,11,12,-,-,-,16,-,-,-	2.08	–	1.19	–	–	0.93
***CYR30***	1,2,3,4,5,6,7,8,9,-,11,12,-,-,-,16,17,-,-	–	–	1.19	7.69	2.63	1.39
***CYR31***	1,2,3,4,5,6,7,8,9,-,11,12,-,14,-,16,17,-,-	4.17	3.03	3.57	7.69	2.63	3.70
***CYR32***	1,2,3,4,5,6,7,8,9,10,11,12,13,14,-,16,17,-,-	4.17	9.09	4.76	7.69	13.16	6.94
***CYR33***	-,2,3,4,5,6,7,8,9,10,11,12,13,14,-,16,-,-,-	4.17	–	3.57	–	–	2.31
***CYR34***	1,2,3,4,5,6,7,8,9,10,11,12,13,14,-,16,17,-,19	6.25	–	7.14	15.38	5.26	6.02
***Guinong22-13***	-,2,3,-,-,6,7,8,9,10,11,12,13,14,-,16,17,-,19	4.17	9.09	4.76	15.38	2.63	5.56
***Guinong22-14***	1,2,3,4,5,6,7,8,9,10,11,12,13,14,15,-,-,-,19	–	–	2.38	–	2.63	1.39
***Hy-4***	1,2,3,4,5,6,7,8,9,10,11,-,13,14,-,16,17,-,-	–	–	1.19	–	–	0.46
***Hy-6***	1,2,3,4,5,6,7,8,9,-,11,-,-,14,-,-,17,-,-	4.17	–	–	–	–	0.93
***Hy-7***	-,2,3,4,5,6,7,8,9,10,11,-,13,14,-,16,17,-,-	2.08	–	–	–	–	0.46
***Lovrin10-2***	1,2,3,-,-,6,7,8,9,-,11,-,-,-,-,16,-,-,-	–	–	1.19	–	–	0.46
***Lovrin13-2***	1,2,3,4,-,6,7,8,9,-,11,12,-,-,-,16,-,-,-	–	–	–	–	5.26	0.93
***Lovrin13-8***	1,2,3,4,5,6,7,8,9,10,11,12,-,-,-,16,-,-,-	2.08	–	–	–	–	0.46
***Nw-1***	-,-,3,-,-,6,-,-,9,-,-,-,-,-,-,-,-,-,-	–	3.03	1.19	–	–	0.93
***Nw-10***	-,2,3,4,5,6,7,-,9,-,11,-,-,14,-,-,17,-,-	–	–	1.19	–	–	0.46
***Nw-11***	1,2,3,4,5,6,7,8,9,10,11,12,13,14,-,16,-,-,-	–	–	1.19	15.38	–	1.39
***Nw-12***	1,2,3,4,-,6,7,8,9,10,11,12,13,14,-,16,-,-,-	–	3.03	–	–	–	0.46
***Nw-13***	-,2,-,4,-,-,7,-,-,-,-,-,-,-,-,-,-,-,-	–	3.03	–	–	–	0.46
***Nw-14***	-,2,3,4,5,6,7,8,9,-,11,-,-,-,-,-,-,-,-	2.08	3.03	–	–	–	0.93
***Nw-15***	-,2,3,4,-,6,7,8,9,10,11,-,13,14,-,16,17,-,-	4.17	–	–	–	–	0.93
***Nw-16***	1,2,3,4,5,6,7,8,-,-,11,-,-,-,-,-,-,-,-	2.08	–	–	–	–	0.46
***Nw-17***	1,2,3,4,5,6,7,-,9,-,11,-,-,14,-,-,17,-,-	2.08	–	–	–	2.63	0.93
***Nw-18***	-,2,3,4,-,6,7,8,-,-,11,-,-,-,-,-,-,-,-	–	–	–	–	2.63	0.46
***Nw-19***	1,2,3,4,-,6,7,8,9,-,11,12,-,14,-,16,-,-,-	–	–	–	–	2.63	0.46
***Nw-4***	-,2,3,-,-,6,7,8,9,-,11,-,-,-,-,-,-,-,-	–	–	1.19	–	–	0.46
***Nw-5***	-,2,3,4,5,6,7,8,-,-,11,-,-,14,-,-,17,-,-	–	–	1.19	7.69	–	0.93
***Nw-6***	1,2,3,4,-,6,7,8,9,10,11,12,-,14,-,16,17,-,-	2.08	–	1.19	–	–	0.93
***Nw-7***	-,2,3,4,-,6,7,8,9,10,11,12,-,14,-,16,17,-,-	–	6.06	–	–	–	0.93
***Nw-8***	-,2,3,4,-,6,7,8,9,10,11,12,13,14,-,16,-,-,-	–	3.03	1.19	–	–	0.93
***Nw-9***	-,2,3,4,5,6,7,8,9,-,11,-,-,14,-,-,17,-,-	–	–	1.19	–	2.63	0.93
***Suwon11-1***	1,2,-,-,-,-,7,-,-,-,-,-,-,14,-,-,-,-,-	6.25	18.18	22.62	–	15.79	15.74
***Suwon11-10***	-,2,3,4,-,6,7,8,9,-,11,-,-,14,-,-,-,-,-	10.42	3.03	5.95	–	10.53	6.94
***Suwon11-12***	-,2,3,-,-,6,7,8,9,-,11,-,-,14,-,16,-,-,-	6.25	3.03	16.67	–	5.26	9.26
***Suwon11-13***	1,2,3,4,-,6,7,8,9,10,11,-,-,14,-,-,-,-,-	6.25	3.03	1.19	–	–	2.31
***Suwon11-2***	1,2,3,4,5,6,7,8,9,-,11,-,-,14,-,-,-,-,-	8.33	12.12	3.57	15.38	7.89	7.41
***Suwon11-3***	1,2,3,4,5,6,7,8,9,10,11,-,13,14,-,-,-,-,-	–	–	–	7.69	–	0.46
***Suwon11-6***	1,2,3,-,-,6,7,8,9,-,11,-,-,14,-,-,-,-,-	4.17	–	–	–	–	0.93
***Suwon11-7***	1,2,3,4,5,6,7,8,9,-,11,12,-,14,-,16,-,-,-	–	3.03	1.19	–	–	0.93
***Suwon11-8***	1,2,3,4,-,6,7,8,9,-,11,-,-,14,-,-,-,-,-	4.17	–	3.57	–	–	2.31
**Total**		**48**	**33**	**84**	**13**	**38**	**216**

a Virulence profile showed race virulence pattern against differential lines: 1 = Trigo Eureka, 2 = Fulhard, 3 = Lutescens 128, 4 = Mentana, 5 = Virgilio, 6 = Abbondanza, 7 = Early Premium, 8 = Funo, 9 = Danish 1, 10 = Jubilejina 2, 11 = Fengchan 3, 12 = Lovrin 13, 13 = Kangyin 655, 14 = Suwon 11, 15 = Zhong 4, 16 = Lovrin 10, 17 = Hybrid 46, 18 = Triticum spelta var. Album, and 19 = Guinong 22.

In the overall population, Race *Suwon 11-1* had the highest frequency (15.74%). This race had a virulent pattern against differentials Trigo Eureka (*Vr1*), Fulhard (*Vr2*), Early Premium (*Vr7*), and Suwon11 (*Vr14*), followed by race *Suwon 11-12* (9.26%). Races *CYR34* (*Vr1, Vr2, Vr3, Vr4, Vr5, Vr6, Vr7, Vr8, Vr9, Vr10, Vr11, Vr12, Vr13, Vr14, Vr16, Vr17*, and *Vr19*) and *CYR32* (*Vr1, Vr2, Vr3, Vr4, Vr5, Vr6, Vr7, Vr8, Vr9, Vr10, Vr11, Vr12, Vr13, Vr14, Vr16*, and *Vr17*) with broad virulence spectra had relative frequencies of 6.02% and 6.94%, respectively (**Table 1**).

**Figure 2:**
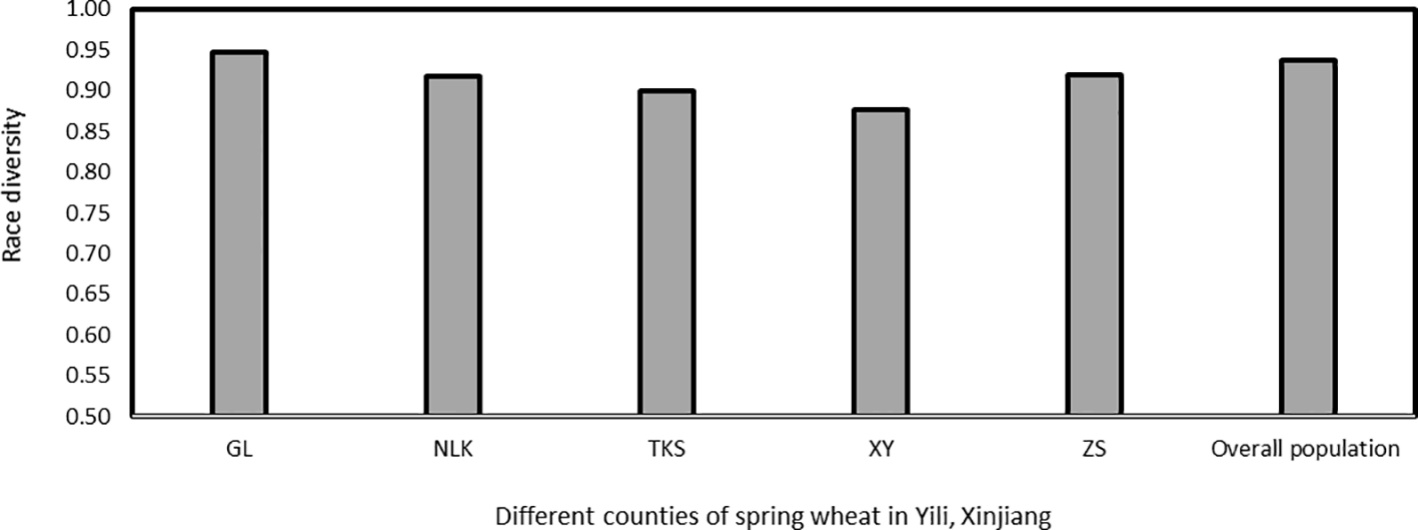
Race diversity in *Puccinia striiformis* f. sp. *tritici* isolates collected from spring wheat, Xinjiang, China in 2021. GL, Gongliu; NLK, Nileke; TKS, Tekesi; XY, Xinyuan; and ZS, Zhaosu.

Race diversities ranged from 0.88 to 0.95 with the highest race diversity observed in the Gongliu counties (**Figure 2**; **Table 2**). In the Gongliu county, the maximum relative frequency was noted by race *Suwon11-10* (10.42%). In Nileke races, *Suwon11-1* (18.18%) and *Suwon11-2* (12.12%) were predominant. In Tekesi, the predominant races were *Suwon11-1* (22.62%) and *Suwon11-12* (16.67%). In Xinyuan, high frequencies were observed for races *CYR34*, *Guinong22-13*, *Nw-11*, and *Suwon11-2* (15.3%). The highest frequency was observed for race *Suwon11-1* in the Zhaosu county.

**Table 2:** Number of races, diversity, and virulences detected in the *Puccinia striiformis* f. sp. *tritici* isolates collected from the spring wheat crop in Xinjiang, China.

Region^a^	No. of isolates	No. of races detected	Race diversity	Frequency of the most frequent race	No. of virulences detected	Frequency of the most frequent virulence
**GL**	48	25	0.95	10.42	17	100.00
**NLK**	33	20	0.92	18.18	17	96.97
**TKS**	84	27	0.90	22.62	18	98.81
**XY**	13	9	0.88	15.38	17	100.00
**ZS**	38	19	0.92	15.79	18	100.00
**Overall population**	216	46	0.94	15.74	18	99.07

a GL, Gongliu; NLK, Nileke; TKS, Tekesi; XY, Xinyuan; and ZS, Zhaosu.

### 3.2 Virulence

**Figure 3:**
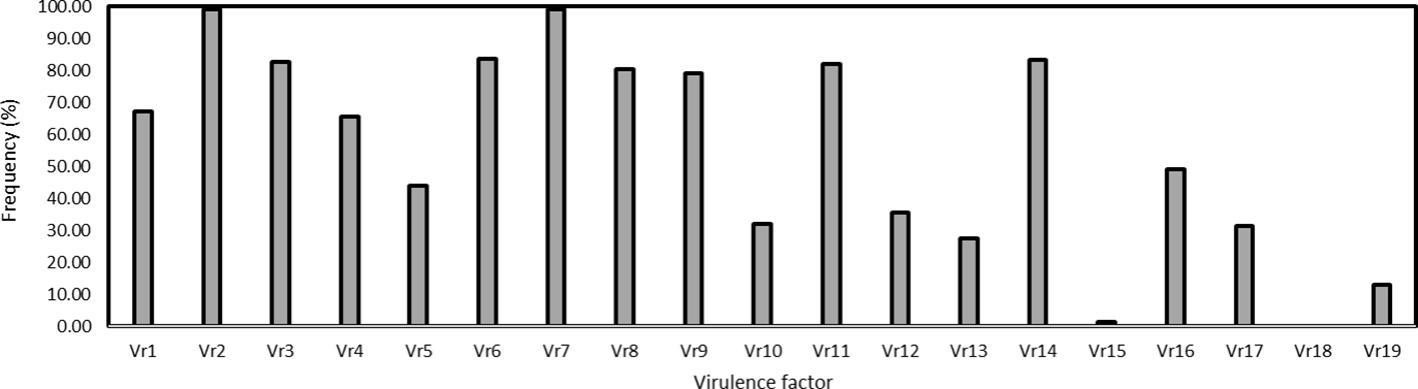
Frequencies of virulence factors detected in the *Puccinia striiformis* f. sp. *tritici* isolates collected from spring wheat crop of Xinjiang, China. The Chinese differentials are as follows: 1 = Trigo Eureka, 2 = Fulhard, 3 = Lutescens 128, 4 = Mentana, 5 = Virgilio, 6 = Abbondanza, 7 = Early Premium, 8 = Funo, C9 = Danish 1, 10 = Jubilejina 2, 11 = Fengchan 3, 12 = Lovrin 13, 13 = Kangyin 655, 14 = Suwon 11, 15 = Zhong 4, 16 = Lovrin 10, 17 = Hybrid 46, 18 = *Triticum spelta* var. Album, and 19 = Guinong 22.

The virulence factors were simply designated as *Vr1*, *Vr2*, … *Vr19* corresponding to the sequential order of the 19 differential lines. Virulence factors *Vr2* and *Vr7*, which overcome the resistance genes in Fulhard and Early Premium, respectively, had greater than 95% frequencies. In contrast, virulence to resistance gene *Yr5* in differential *Triticum spelta* var. Album was not detected. Also, virulence to differential Zhong 4 had a low frequency (**Table 3**; **Figure 3**).

**Table 3:** Frequencies (%) of virulence factors in *Puccinia striiformis* f. sp. *tritici* isolates collected from spring wheat crops in Xinjiang, China.

Virulence ^a^	Region ^b^					Overall population
	GL	NLK	TKS	XY	ZS	
***Vr1***	66.67	66.67	61.90	76.92	76.32	67.13
***Vr2***	100.00	96.97	98.81	100.00	100.00	99.07
***Vr3***	93.75	75.76	77.38	100.00	81.58	82.87
***Vr4***	79.17	63.64	51.19	84.62	76.32	65.74
***Vr5***	47.92	33.33	38.10	84.62	47.37	43.98
***Vr6***	93.75	78.79	77.38	100.00	84.21	83.80
***Vr7***	100.00	96.97	98.81	100.00	100.00	99.07
***Vr8***	91.67	72.73	75.00	100.00	78.95	80.56
***Vr9***	89.58	72.73	76.19	92.31	73.68	79.17
***Vr10***	35.42	33.33	28.57	61.54	23.68	31.94
***Vr11***	93.75	72.73	76.19	100.00	81.58	81.94
***Vr12***	29.17	36.36	33.33	69.23	36.84	35.65
***Vr13***	25.00	24.24	26.19	61.54	23.68	27.31
***Vr14***	83.33	75.76	89.29	92.31	73.68	83.33
***Vr15***	0.00	0.00	2.38	0.00	2.63	1.39
***Vr16***	43.75	42.42	53.57	69.23	44.74	49.07
***Vr17***	33.33	27.27	27.38	61.54	31.58	31.48
***Vr18***	0.00	0.00	0.00	0.00	0.00	0.00
***Vr19***	10.42	9.09	14.29	30.77	10.53	12.96
**Sample size**	**48**	**33**	**84**	**13**	**38**	**216**

a Different virulent (Vr) corresponding differential lines: 1 = Trigo Eureka, 2 = Fulhard, 3 = Lutescens 128, 4 = Mentana, 5 = Virgilio, 6 = Abbondanza, 7 = Early Premium, 8 = Funo, C9 = Danish 1, 10 = Jubilejina 2, 11 = Fengchan 3, 12 = Lovrin 13, 13 = Kangyin 655, 14 = Suwon 11, 15 = Zhong 4, 16 = Lovrin 10, 17 = Hybrid 46, 18 = Triticum spelta var. Album, and 19 = Guinong 22.

b Regions GL = Gongliu, NLK = Nileke, TKS = Tekesi, XY = Xinyuan, and ZS = Zhaosu.

### 3.3 Comparison of predominant races in the winter and spring wheat regions

**Figure 4:**
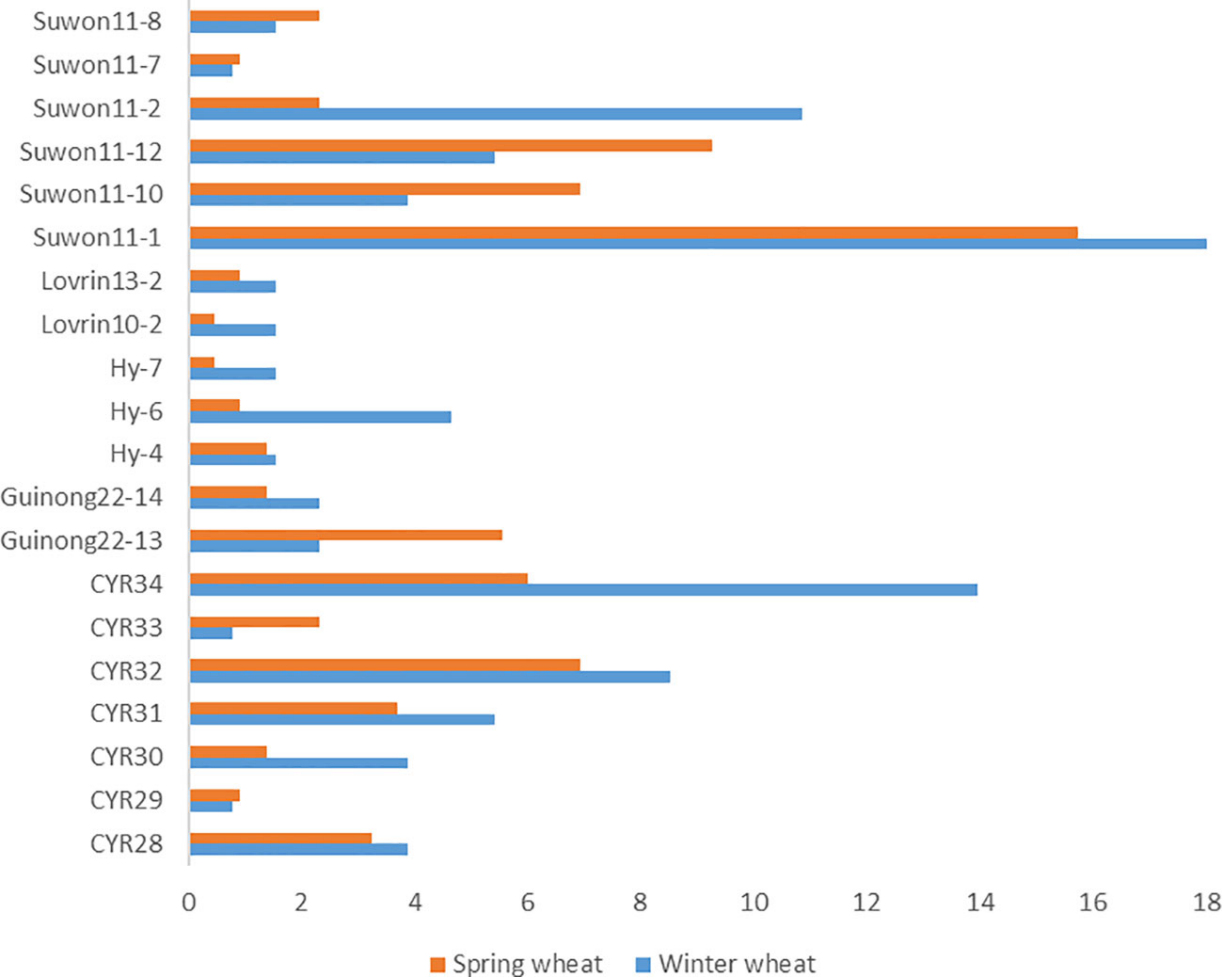
*Puccinia striiformis* f. sp. *tritici* races commonly detected from the winter and spring wheat regions of Xinjiang, China in 2021.

We compared races identified from the spring wheat region with the study on races identified in the winter wheat crop in the same year in Xinjiang (Chen et al., 2023). The predominant races identified from winter wheat, such as *Suwon 11-1*, *Suwon11-2*, *Suwon 11-17*, *Loverin10-2*, *Hy-6*, *Guinong22-14*, *CYR34*, *CYR32*, *CYR30*, and *CYR28*, were also identified from spring wheat (**Figure 4**). The most predominant race in both winter wheat and spring wheat crops was *Suwon11-1*. Some races had big differences in frequencies between the crop regions. For example, races Hy6, CYR34, and CYR30 had much higher frequencies in the winter wheat crop than the spring wheat crop. The race diversity was higher in spring wheat than in winter wheat.

## 4 Discussion

Wheat crop is severely affected by stripe rust worldwide. Monitoring the disease and identifying races of the pathogen help researchers develop resistant cultivars. Understanding the dynamics of races within different host crops and seasons is vital to break up the regional pathogen life cycle. Xinjiang is one of the most important epidemic regions for wheat stripe rust not only for China but also for Central Asian countries due to its geographic proximity (Awais et al., 2022; Chen et al., 2023). Very limited research was conducted on race identification in this region. Recently, Chen et al. (2023) identified races from the winter wheat region but isolates from spring wheat were not included. In the present study, we obtained 216 isolates from spring wheat crops from Xinjiang and identified 46 races.

Our results showed that race *Suwon11-1* dominated the overall spring population, which was also the most predominant race in the same year in the winter wheat crop of Xinjiang (Chen et al., 2023). In addition, races *Suwon 11-2. Suwon 11-17, Loverin10-2, Hy-6, Guinong22-14, CYR34, CYR32, CYR30*, and *CYR28* were also found in both crops. This suggests that urediniospores produced on the winter wheat crop can carry over to the spring wheat crop, and vice versa. Also, barberry species near wheat fields may produce new races through sexual reproduction (Zhuang et al., 2019). Recently, stripe rust found on volunteer wheat and various grasses during our field surveys (unpublished data) suggested that volunteer wheat and grasses may serve as the host bridges for *Pst* survival and transfer from late-maturing winter and spring wheat to autumn-sown winter wheat seedlings (Zeng et al., 2022).

Race identification from neighboring countries has not yet been done to understand whether Xinjiang plays any role in inter-regional migration, but it needs to be studied. The *Su11* race group that was virulent to Chinese differential Suwon 11 was dominant in China during 1994–1996 (Zhao and Kang, 2023) and was recently reported in Xinjiang in a previous study (Zhan et al., 2016). In the present study, we found that some of the *Su11* race group members were still predominant. These results suggest that the *Su11* race group has a high fitness, well adapting to the wheat cultivars and environments in Xinjiang. Although at relatively low frequencies, the recently identified races like CYR33 and CYR34 were detected in the present study and in the winter wheat region of Xinjiang (Chen et al., 2023). More importantly, we identified 17 new races with the virulence profiles that have not been found in any other regions of China. Continuous monitoring of the disease and the pathogen races in the vast Xinjiang region is needed for the effective control of stripe rust in this region and other regions of China.

The resistance genes in Fulhad and Early Premium are ineffective against the Pst population in Xinjiang and the other regions of China. In contrast, *Yr5* is still effective. Similar results were also found in the winter wheat region of Xinjiang (Chen et al., 2023). Rotations of host and non-host crops in different seasons may help farmers to reduce stripe rust damage (Bargués-Ribera and Gokhale, 2020). However, growing resistant cultivars is the most effective approach to control the diseases. Selecting resistant wheat cultivars from currently available ones in the region is the immediate step, but developing new cultivars with improved stripe rust resistance is an urgent and long task. Breeders should consider all race groups for developing wheat cultivars with effective resistance. It should be better to use various combinations of effective race-specific resistance genes with genes for durable resistance to develop wheat cultivars with adequate and long-lasting resistance. Also, collaborations within and beyond the Xinjiang region in stripe rust monitoring, research, and breeding are necessary as the region is huge and the pathogen is capable of the long-distance migration.

## 5 Conclusions

This study conducted surveillance from the spring wheat region to identify the *Puccinia striiformis* f. sp. *Tritici* races. A total of 46 *Pst* races were identified, namely, 29 previously identified races and 17 new races from 216 isolates. Races *Suwon-11-1*, *Suwon11-12*, and *CYR32* had high frequencies in the spring wheat region. The predominant races in winter wheat in the same season were also detected from spring wheat cultivars, indicating Pst spreading from winter wheat to spring wheat crops. Deploying resistance genes and regular race identification from that region is vital to overcome future epidemic threats.

## Data availability statement

The original contributions presented in the study are included in the article/**Supplementary Material**, further inquiries can be directed to the corresponding authors.

## Author contributions

JM: Conceptualization, Formal Analysis, Investigation, Writing – original draft. MA: Formal Analysis, Writing – original draft. LC: Investigation, Writing – original draft. HY: Investigation, Validation, Writing – original draft. HL: Investigation, Writing – original draft. YS: Investigation, Writing – original draft. HW: Investigation, Writing – original draft. GL: Conceptualization, Methodology, Writing – original draft. HG: Conceptualization, Formal Analysis, Investigation, Writing – original draft.
