# Supplementary material for: Identification of Puccinia striiformis races from the spring wheat crop in Xinjiang, China
Source: Front Plant Sci. 2023 Oct 3;14:1273306. doi: 10.3389/fpls.2023.1273306 (PMC10586046; doi:10.3389/fpls.2023.1273306)
Supplement: Supplementary file 2 [file Table_2.docx]

**Table 1: Chinese differential lines used to identify races of *Puccinia striiformis* f. sp. *tritici* isolates collected from spring wheat, Xinjiang, China, in 2021**

|  | **Differential**  **Order** |  | **Differential lines** | **Yr gene^a^** |
| --- | --- | --- | --- | --- |
|  | 1 |  | Trigo-Eureka | *Yr6* |
|  | 2 |  | Fulhard | Unknown |
|  | 3 |  | Lutescens 128 | Unknown |
|  | 4 |  | Mentana | Unknown |
|  | 5 |  | Virgilio | *YrVir1*, *YrVir2* |
|  | 6 |  | Abbondanza | Unknown |
|  | 7 |  | Early Premium | Unknown |
|  | 8 |  | Funo | *YrA*,*+* |
|  | 9 |  | Danish 1 | *Yr3* |
|  | 10 |  | JubilejinaII | *YrJu1*, *YrJu2*, *YrJu3*, *YrJu4* |
|  | 11 |  | Fengchan 3 | *Yr1* |
|  | 12 |  | Lovrin 13 | *Yr9*,*+* |
|  | 13 |  | Kangyin 655 | *Yr1*, *YrKy1*, *YrKy2* |
|  | 14 |  | Suwon 11 | *YrSu* |
|  | 15 |  | Zhong 4 | Unknown |
|  | 16 |  | Lovrin 10 | Yr9 |
|  | 17 |  | Hybrid 46 | *Yr3b*, *Yr4b* |
|  | 18 |  | *Triticum spelta* var. *Album* | *Yr5* |
|  | 19 |  | Guinong22 | *Yr10*, *Yr26* |

^a^The *Yr* genes in differentials lines refer to Wan et al. [27] : Chen et al. [35] and Zhan et al. [16] .
